# Early Dermoscopic Signs Leading to Primary Systemic Amyloidosis’ Diagnosis: A Case Report

**DOI:** 10.5826/dpc.1103a23

**Published:** 2021-07-08

**Authors:** Trashita Hassanandani, Bhabani S.T.P. Singh, Bikash Ranjan Kar

**Affiliations:** Department of DVL, IMS & SUM Hospital, Bhubaneswar, Odisha, India

**Keywords:** Dermoscopy, primary systemic amyloidosis, cutaneous

## Introduction

Amyloidosis is characterised by an extracellular deposition of insoluble polymeric protein fibrils in tissues and organs. Skin and mucosae can be involved either as a part of primary cutaneous amyloidosis or secondary to systemic amyloidosis. Cutaneous changes are seen in about 25% of patients with primary systemic amyloidosis. Here we present unique dermoscopic features of cutaneous lesions in a case of Primary Systemic Amyloidosis (PSA).

## Case Presentation

**Figure 1:**
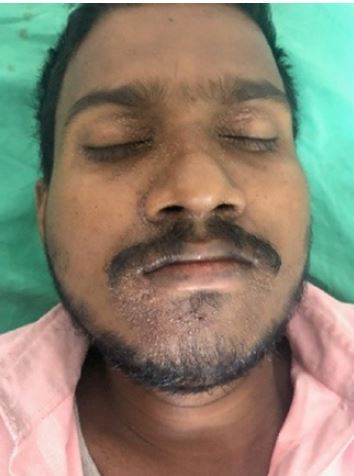
Skin to reddish coloured papules around the eyes, nose, and lips.

A 26-year-old male presented closely set skin-coloured papules over the eyelids, around the nose, mouth, and over the scalp. Few papules had turned red over the past 6 months (Figure 1).

**Figure 2:**
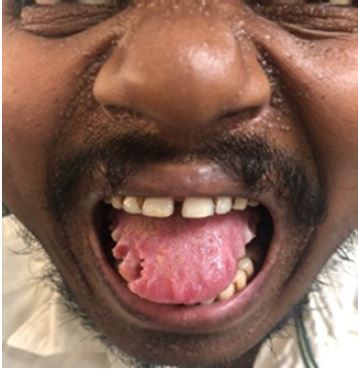
Macroglossia with teeth indentation marks on the sides of the tongue

**Figure 3:**
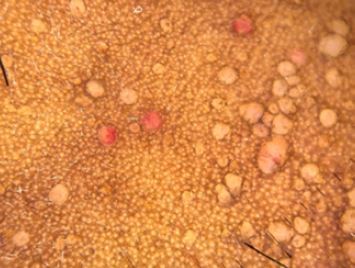
White to yellowish background with few lesions showing glomerular vessels (DermLite DL3, polarized view).

**Figure 4:**
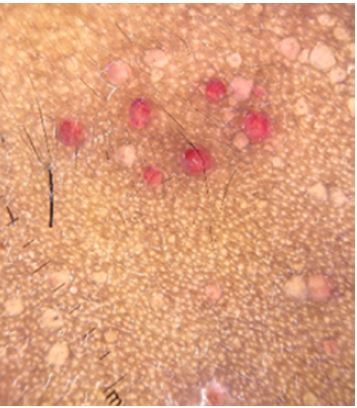
Red to pinkish background with vessels. (DermLite DL3, polarized view).

The patient reported a history of generalized weakness, hoarseness of voice, and an increase in the size of his tongue (Figure 2). Dermoscopic analysis revealed lesions characterized by white to yellowish clods. Many lesions showed vascular proliferation with a glomerular pattern against a red to pink background (Figures 3 and 4).

**Figure 5:**
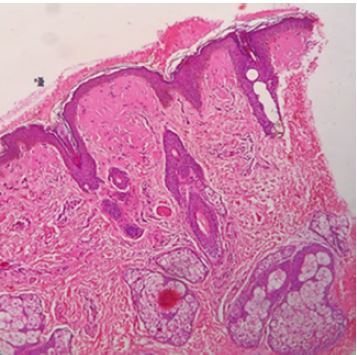
Histopathological features showing clumps of pale acellular eosinophilic material in the papillary dermis. (H&E, ×10).

**Figure 6:**
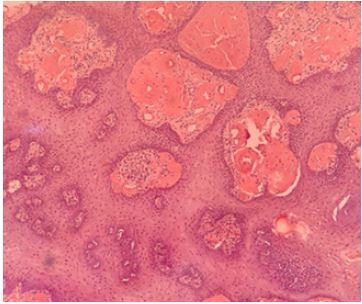
Congo red stain shows brick-red coloured amyloid deposits in the dermis and around blood vessels.

Histopathology showed clumps of pale acellular eosinophilic material (Figure 5), positive for Congo red stain in the papillary dermis (Figure 6).

Further Investigations revealed renal impairment with proteinuria. 24-hour urine protein was 1879.8 mg/day. Urine electrophoresis showed monoclonal spikes in the gamma region. A final diagnosis of myeloma associated systemic amyloidosis was made.

The patient started Bortezomib-Cyclophosphamide-Dexamethasone treatment cycles every 28 days and is currently under follow up.

The most common clinical cutaneous presentations of systemic amyloidosis are haemorrhagic lesions (petechiae, purpura, and ecchymoses), which are caused by amyloid deposition within dermal blood vessel walls, leading to vessel fragility. When there is a greater amount of amyloid deposition in the skin, the patient may develop papules, plaques, and even nodules, which may exhibit a haemorrhagic appearance.

In our case, the lesions were mainly translucent to skin coloured. Dermoscopic findings showed white to yellowish clods. Vascular proliferation was distinctly seen in a glomerular pattern with a red to pink background. The yellowish appearance of skin lesions on dermoscopic examination may correspond histologically to amyloid deposition in the upper dermis. The glomerular vessels appearing as clustered or coiled vessels located on one side of the lesion resembling renal glomeruli, correspond possibly histologically to small dermal blood vessels. A dermoscopic examination can detect early vascular changes, prior to the clinical appearance of classically evident haemorrhagic papules of systemic amyloidosis. In literature, these glomerular vessels are commonly encountered in non-pigmented Bowen’s disease and superficial basal cell carcinoma. Nevertheless, there are no reports of these vascular patterns in amyloidosis. In a report by Hu et al (2019), dermoscopy of facial papules in a case of systemic amyloidosis revealed a diffusely yellowish surface with pinpoint petechiae [1]. A high degree of suspicion and early ular pattern with a red to pink background. The yellowish appearance of skin lesions on dermoscopic examination may correspond histologically to amyloid deposition in the upper dermis. The glomerular vessels appearing as clustered or coiled vessels located on one side of the lesion resembling renal glomeruli, correspond possibly histologically to small dermal blood vessels. A dermoscopic examination can detect early vascular changes, prior to the clinical appearance of dermoscopic evaluation can help prompting systemic amyloidosis’ diagnosis with cutaneous involvement [2].

## Conclusion

Glomeruloid vessels found in a yellowish background can represent a clinical feature of cutaneous lesions of primary systemic amyloidosis. These cutaneous findings can serve as an early dermoscopic sign to suspect cutaneous changes before the classic haemorrhagic appearance of systemic amyloidosis.
